# Targeting Postprandial Hyperglycemia With Physical Activity May Reduce Cardiovascular Disease Risk. But What Should We Do, and When Is the Right Time to Move?

**DOI:** 10.3389/fcvm.2018.00099

**Published:** 2018-07-18

**Authors:** Thomas P. J. Solomon, Frank F. Eves, Matthew J. Laye

**Affiliations:** ^1^School of Sport, Exercise, and Rehabilitation Sciences, College of Life and Environmental Sciences, University of Birmingham, Edgbaston, United Kingdom; ^2^Institute of Systems and Metabolism Research, College of Medical and Dental Sciences, University of Birmingham, Edgbaston, United Kingdom; ^3^Department of Health and Human Performance, College of Idaho, Caldwell, ID, United States

**Keywords:** exercise, inactivity, sedentary behavior, sitting time, standing, walking, Type 2 diabetes, motivation

## Abstract

Physical inactivity and excessive postprandial hyperglycemia are two major independent risk factors for type 2 diabetes and cardiovascular-related mortality. Current health policy guidelines recommend at least 150 min of physical activity per week coupled with reduced daily sedentary behavior by interrupting prolonged sitting with bouts of light activity every 30-min. This evidence-based strategy promotes health and quality of life. Since modern lifestyle enforces physical inactivity through motorized transportation and seated office working environments, this review examines the practical strategies (standing, walking, stair climbing, and strength-based circuit exercises) for reducing sitting time and increasing activity during the workday. Furthermore, since postprandial hyperglycemia poses the greatest relative risk for developing type 2 diabetes and its cardiovascular complications, this review examines a novel hypothesis that interrupting sitting time would be best focused on the postprandial period in order to optimize blood glucose control and maximize cardiometabolic health. In doing so, we aim to identify the science gaps which urgently need filling if we are to optimize healthcare policy in this critical area.

## Introduction

Since Plato wrote, “And is not the bodily habit spoiled by rest and idleness, but preserved for a long time by motion and exercise?” (1), it is now an age-old message that physical inactivity is a cause of several chronic conditions. In 2012, the Global Burden of Diseases, Injuries and Risk Factors Study found low physical activity to be the fourth leading cause of global mortality, ahead of being overweight/obese; physical inactivity was estimated to contribute to ~1 in 10 premature deaths from coronary heart disease (2). For this reason, the UK Department of Health currently recommends all adults to undertake at least 150 min of moderate intensity exercise each week (or 75 min of vigorous intensity), combined with muscle-strengthening activities on at least 2 days per week, and to minimize the amount of time spent being sedentary (sitting) for extended periods. However, despite advances in scientific knowledge and large-scale media attention, in 2016 the Health Survey for England found that adults in the UK spend ~5 h per day sitting in their spare time (not including hours at paid work) and that only 26% of adults over 19 meet the above-described activity recommendations (3). Such questionnaire-based epidemiological assessments of activity levels are vast overestimates. The Health Survey for England in 2008 objectively measured physical activity levels using accelerometers, finding that only ~5% (vs. ~35% from self-report questionnaires) of the UK achieved physical activity guidelines (4). Similar data has emerged from the U.S. where the 2005–2006 National Health and Nutrition Examination Survey (NHANES) found that only ~3.2% of U.S. adults achieved physical activity guidelines, which are similar to the UK (5). In 2012, Ng and Popkin published a comprehensive historical analysis of physical activity levels and sedentary behavior (e.g., sitting time) in five populous nations: the US (1965 to 2009, the UK (1961 to 2005), China (1991 to 2009), Brazil (2002 to 2007), and India (2000 to 2005). The picture was clear: physical activity levels are declining and sedentary behavior is increasing (6). Because the majority of physical inactivity is derived from daily sitting time, this review will discuss appropriate and feasible physical activities that may be used to interrupt and prevent prolonged sitting. This review will also discuss the optimal timing between meals and interruption of sitting time (through different physical activity approaches) in the context of preventing poor postprandial blood glucose control, the hyperglycemic phenotype of diabetes that is a major contributor to cardiovascular-related mortality (7).

## Type 2 diabetes and cardiometabolic health—how is inactivity implicated and why does physical activity matter?

Diabetes is a major health problem because it causes blindness, kidney failure, and lower limb amputations, and between 1980 and 2014 global prevalence doubled to 8.5%, equivalent to 422 million people (8). People with diabetes are also 2 to 3-times more likely to die of a cardiovascular event (7). Ultimately, diabetes drastically reduces quality of life and longevity, and places a huge economic burden on health care systems. Type 2 diabetes is characterized by persistent hyperglycemia caused by insufficient insulin secretion to compensate for poor insulin sensitivity. The disease is indicated when poor blood glucose control is detected with clinical tests for glycated hemoglobin (HbA1c), fasting glucose, or 2-h glucose during an oral glucose tolerance test (OGTT) (9). Prevention and treatment of type 2 diabetes involves a multifaceted approach including lifestyle modification, nutritional counseling, and pharmacological therapy (10). At the core of the lifestyle modification, are physical activity guidelines recommending that adults with diabetes should engage in ≥150-min of moderate-to-vigorous intensity aerobic exercise (or ≥75-min of vigorous or interval training) and 2–3 resistance exercise sessions per week, with no more than two consecutive days without activity (11). An additional recommendation is that sedentary behavior should be decreased by interrupting prolonged sitting every 30-min (11). Although these standards of care are almost identical to recommendations made for all adults, they are informed by published evidence. For example, the Nurses' Health Study examined type 2 diabetes incidence by measuring fasting and/or 2-h glucose during OGTT in a 6-year follow-up in ~70,000 individuals. Each 2-h/day increment in time spent watching TV or time spent sitting at work was associated with a 14 and 7% increased risk of diabetes, respectively (12). The same study also found that 2-h/day of standing or walking at home was associated with a 12% reduction in diabetes whereas brisk walking for 1-h/day was associated with a 34% reduction in diabetes (12). Experimental evidence from randomized controlled trials also demonstrate that the standard of care activity guidelines help maintain good blood glucose control in nondiabetic individuals (13) and help prevent deterioration of blood glucose control in patients with diabetes (14, 15). Since diabetes is a major risk for cardiovascular disease and mortality (7), the physical activity guidelines are also useful for optimizing cardiometabolic health. Similarly, sedentary behavior (sitting time) and low physical activity levels are strongly associated with increased cardiovascular disease risk and cardiovascular-related mortality in major studies like the Nurses' Health Study, the Lancet Sedentary Behavior Working Group, and the 45 and Up Study (12, 16–23). Therefore, as of 2018, these aforementioned epidemiological and experimental studies provide clear evidence to support the use of physical activity as a therapeutic modality for reducing diabetes and cardiometabolic risk. Given that ~3% of people are meeting activity guidelines [based on accelerometer data from the UK and US (4, 5)], it is not surprising that type 2 diabetes continues to affect nearly 10% of the global population (8). With such a low attainment of physical activity guidelines, we are far from implementing the full potential of the beneficial health stimulus physical activity can provide. A more reasonable goal, therefore, might be first to interrupt sedentary time with physical activity.

## What is the impact of interrupting sitting time on type 2 diabetes risk and cardiometabolic health?

Although physical activity guidelines have long been available, the addition of inactivity guidelines to manage sedentary behavior by reducing sitting time is relatively new. For example, the recommendation to interrupt prolonged sitting was added to the American Diabetes Association's standards of care for diabetes in 2017 (24). More recently, experimental studies during single days have investigated the direct effects of interruption to inactivity (sitting time) on glucose control. The first of these by Dunstan and colleagues in 2012 showed that interrupting a 5-h period of sitting with 2-min of light or moderate walking every 20-min significantly reduced the blood glucose response to a mixed-nutrient liquid meal, in 19 overweight middle-aged individuals (25). Similarly, it was also found that regular treadmill walking breaks (1 min 40 s every 30 min) during 9-h of sitting significantly reduced the blood glucose response to mixed-nutrient meals during the day, in 70 young, lean, healthy individuals. In this work, the interruption protocol had a greater effect than a single 30-min walk (26). Such findings have been confirmed by Bailey and colleagues who found that interrupting sitting for 4-h following a mixed-nutrient breakfast with a 2-min treadmill walk every 20 min attenuated the postprandial increase in blood glucose in 14 young adults (27). These single-day observations have been extended to over 4-days by Duvivier and colleagues who studied 18 young, lean, healthy individuals in whom 14-h of sitting was interrupted with either 1-h of vigorous cycling or 4-h of walking and 2-h of standing (28). After the 4th day, neither fasting glucose nor AUC glucose during OGTT were different between groups while insulin sensitivity increased in the walking/standing group but not in the vigorous exercise group. This suggests that reducing inactivity with more time spent walking/standing is more effective than 1-h of vigorous exercise (28). In 2017, the same authors repeated this study in 19 older patients with type 2 diabetes using continuous glucose monitoring to assess 24-h glucose control during the 4-day interventions. Their findings showed that 24-h glucose AUC and time spent hyperglycemic (>10 mM) were significantly reduced by sitting less with 2-h of walking plus 3-h of standing, but not by ~1-h of cycling (29), again highlighting the effectiveness of replacing sedentary behavior with light physical activity, such as walking, in maintaining glucose control.

Further studies have examined the effects of simply standing to interrupt prolonged sitting. Epidemiological observations indicate that increased standing to break up sitting is protective against cardiovascular (30) and all-cause (31) mortality, but some detailed intervention studies have also been conducted. Henson and colleagues objectively compared standing vs. walking at ~3 km per h as means of interrupting 7.5-h of sitting for 5-min every 30-min, during a single-day in 22 overweight, middle-aged women. They found that standing or walking elicited the same improvement in blood glucose AUC following mixed-nutrient meal ingestion (32). Similarly, daylong postprandial glucose responses are reduced by interrupting daylong sitting with either standing, walking, or cycling during the day, in 9 overweight/obese adults (33). Buckley and colleagues also found lower AUC glucose (via continuous glucose monitoring) during 185-min of standing vs. sitting while individuals worked in an office environment in a non-crossover design of 10 people (34). Furthermore, direct comparisons between interstitial glucose derived from CGM in this study with plasma glucose measurements made in other studies should be made with caution. On the contrary, in a pooled analysis of three trials in 9 overweight, middle to older aged adults, another study found that regular standing breaks (2-min every 20-min) during a 5-h sit was insufficient to improve postprandial glucose control (35). In contrast, the same study found that light- and moderate-intensity walking caused progressively larger improvements in blood glucose AUC following mixed-nutrient meal ingestion (35). These findings are important since they indicate that activities that increase energy expenditure more so than just standing still may be required to optimize glucose control. Indeed similar observations have also been documented during a single day in younger and healthier weight adults (36–38). A contrary hypothesis is that longer-term standing interventions are required to improve blood glucose control. For example, in 2014 it was found that 5-days of alternating between sitting and standing every 30 min during the work day significantly reduced postprandial area under the glucose curve in overweight/obese sedentary office workers, compared to 5-days of prolonged sitting (39).

From the evidence presented above, it is clear that interruption of prolonged sitting with walking or cycling or even just standing up may be effective for improving postprandial blood glucose control, an independent cardiometabolic risk factor. However, as of 2018, no long-term large-scale experimental study has determined the effect of interrupting sitting time on diabetes risk and/or hard endpoints like subsequent cardiovascular complications and mortality.

## When is the right time to use physical activity to interrupt sitting in order to maximize blood glucose control and cardiometabolic health?

Epidemiological and experimental studies show that the degree to which blood glucose is elevated 1–2-h following a meal is associated with increased cardiovascular disease risk (40). Furthermore, evidence also shows that postprandial hyperglycemia in well-controlled diabetes patients is the predominant contributor to elevated levels of glycated hemoglobin (HbA1c), the gold standard biomarker of mean glucose control over the prior 6–8 weeks (41). Accordingly, management of postprandial hyperglycemia is highly prudent particularly given that people spend a large proportion of the day in a postprandial state, and given that diabetes patients are hyperglycemic (>10 mmol/L) for up to 24% of their day (42).

The studies described in the previous section provide evidence that interruption of prolonged sitting with physical activity may improve postprandial blood glucose control. However, such studies did not examine the timing between physical activity and meals and, as of 2018, no long-term randomized, controlled physical activity or exercise intervention study with a primary focus on blood glucose control has reported activity-meal timing. Whether activity/exercise is completed in the fed or fasted state is seldom reported in training studies and as authors of prior studies in this field, we too are guilty. Sometimes this information is not known since activity interventions are self-administered, but activity-meal timing information is even lacking in studies where bouts of activity have been fully-supervised.

Postprandial blood glucose levels are determined by several factors, such as the total caloric value of a meal, macronutrient composition, and carbohydrate quality (e.g., glycemic index/load), all of which may be monitored and controlled. However, multiple other factors are more complex because they cannot be controlled and are variable between individuals. These include gastric emptying rate, intestinal absorption rate, enteroendocrine incretin secretion, incretin sensitivity, pancreatic beta-cell insulin secretory function, hepatic insulin extraction, hepatic glucose production, glucose effectiveness, glucose uptake in all tissues (especially brain, adipose, liver, muscle), insulin sensitivity, and renal glucose reabsorption. An abundance of studies (far too many to cite) also show that the above factors are also altered by a single bout of exercise and/or changed following a prolonged period of increased physical activity or structured exercise training. That said, large inter-individual variability exists in the hyperglycemia-lowering effect of physical activity in individuals with prediabetes or type 2 diabetes (13, 43, 44). For example, HbA1c improved in only two-thirds of patients enrolled in a 3–4-month training intervention (43) while 42% of participants in the HERITAGE study showed no improvement or a deterioration in insulin sensitivity (13). One plausible source of such variability is activity-meal timing.

In 2014, Elsamma Chacko published a letter stating that mid-postprandial moderate-intensity activity (commencing 30-min post-ingestion and lasting up to an hour) is the best time for lowering postprandial hyperglycemia (45). This suggestion was derived from the author's own anecdotal experiences as a medical doctor living with type 2 diabetes in combination with evidence from the very few published experimental studies. Fed vs. fasted exercise has been examined in the context of VO_2_max and/or fat oxidation for optimizing athletes' performance in hundreds of publications. A large number of studies have also studied the interactions between exercise-timing, insulin dosing, and carbohydrate intake for managing blood glucose and preventing hypoglycemia in patients with type 1 diabetes. However, there is a relative paucity of data comparing fed vs. fasted physical activity in relation to type 2 diabetes and cardiometabolic risk. One example from 2001 found that in 10 middle-aged men with type 2 diabetes blood glucose levels were significantly lower when 60-min of moderate-intensity bicycle ergometry were completed 2-h after breakfast, rather than before breakfast (46). While a non-activity control intervention was not included, postprandial plasma free fatty acids were reported but not different between trials. Further work by Colberg and colleagues found that when 12 older-aged, obese, men and women with type 2 diabetes completed 20-min of self-paced treadmill walking starting 15–20 min after eating dinner, blood glucose was significantly lowered from baseline when compared to the pre-dinner walking or no walking (47). However, daylong blood glucose levels show a different pattern: Borer and colleagues found that daylong blood glucose levels were significantly lower when two 2-h low-intensity treadmill walks were completed pre-meal compared to post-meal walking, in 9 overweight, middle to older aged women (48). That said, subjects had noticeable hypoglycemia following the first meal, while no differences in postprandial plasma free fatty acid levels were noted between the pre- or post-meal exercise groups. Another experimental design compared pre- vs. post-breakfast treadmill walking (60-min of continuous moderate intensity or intervals of 1-min hard/3 min easy) to a non-walking control group in 10 older aged, overweight/obese, men and women with type 2 diabetes (49). Similarly, pre-breakfast exercise was more effective at lowering total postprandial hyperglycemia during the day than post-breakfast exercise (49). Moreover, the reduction in total postprandial hyperglycemia during the day was equal between interval- and continuous-walking groups. However, interval walking significantly lowered post-breakfast AUC glucose compared to no exercise and was more effective than continuous walking (49). In addition to aerobic exercise, other work also examined resistance exercise-meal timing in 13 middle-aged, obese, men and women with type 2 diabetes (50). Pre- and post-meal resistance exercise equally improved blood glucose AUC following dinner regardless of timing. However, postprandial triglycerides were significantly lower in the post-meal exercise group, suggesting that post-dinner resistance exercise may more effectively improve cardiometabolic health in patients with diabetes since it lowers both postprandial hyperglycemia and dyslipidemia.

Retrospective analyses of patient food and activity logs from a 12-week training study including three supervised 60-min sessions/week of moderate intensity bicycle ergometry, in 19 middle-aged, overweight men with type 2 diabetes, provided insight into the optimal activity-meal timing (51). Again, blood glucose levels were significantly decreased when cycling was initiated following ingestion of meals but not when cycling was completed in the fasting state. The amount of time after a meal may also be important. Another retrospective study where 15 older aged, obese, men and women with type 2 diabetes completed five 30- to 60-min supervised exercise sessions/week for 12-weeks, found significantly greater reductions in blood glucose when meals were ingested less than 2-h prior to exercise, rather than more than 2-h (52). However, neither a control group nor post-meal exercise data were included.

The above-described literature addressing activity-meal timing is indeed scant with small sample sizes and dichotomous outcomes (Table 1). Some studies are retrospective and some do not include a non-activity control group. Despite current efforts to elucidate the optimal exercise-meal timing, a prospective randomized controlled trial that thoroughly assesses the time course of activity-meal timing on postprandial hyperglycemia and other cardiometabolic risk factors (such as postprandial lipemia) is urgently required. Such an intervention should also separately examine nondiabetic individuals and people with type 2 diabetes, in order to inform guidelines for diabetes prevention as well as diabetes treatment. Since carbohydrate quality influences postprandial glycemia and has been shown to influence exercise adaptations for some (53) but not all (54) variables, examination of the timing between exercise and meals of differing glycemic index/load is also prudent. Besides walking, other practical means to interrupt sitting time, such as standing, stair climbing, or body-weight circuit exercises, also remain to be investigated in the context of an activity-meal time course. Given the different effects of pre- vs. post-meal activity on blood glucose control, the lack of exercise-induced improvement in blood glucose control documented in some studies (55–58) may have been influenced by activity-meal timing. To enhance knowledge, it is prudent for future training studies to consider and report activity-meal timing.

**Table 1:** A summary of published studies that have examined the effect of pre-meal vs. post-meal physical activity on postprandial glycemia and lipemia.

**Study**	**Prospective or retrospective analysis?**	**Subjects**	**Activity**	**Did the study include a no-activity control group?**	**Was post-meal activity better than pre-meal activity?**	
					**Glucose**	**Lipids**
Poirier et al. (46)	Prospective	10 middle-aged men with type 2 diabetes	60 min moderate-intensity cycling	No	Yes Postprandial activity caused lower postprandial blood glucose response than preprandial activity	Not different Postprandial plasma FFA response was not different between pre- vs. post-prandial activity trials
Poirier et al. (51)	Retrospective	19 middle-aged, overweight men with type 2 diabetes	Three 60 min moderate-intensity cycling sessions per week for 12 weeks	No	Yes Postprandial activity caused lower postprandial blood glucose response than preprandial activity	Not measured
Colberg et al. (47)	Prospective	12 older-aged, obese, men and women with type 2 diabetes	20 min self-paced walk	Yes	Yes Postprandial activity caused lower postprandial blood glucose response than preprandial activity and no activity	Not measured
Borer et al. (48)	Prospective	9 overweight, middle to older aged women	Two 2-h low-intensity treadmill walks in a single day, 5-h apart	No	No Pre-meal activity caused lower daylong blood glucose levels than post-meal walking	Not different Postprandial plasma FFA response was not different between pre- vs. post-prandial activity trials
Terada et al. (52)	Retrospective	15 older aged, obese, men and women with type 2 diabetes	Five 30–60 min cycling or walking sessions per week for 12 weeks (continuous moderate intensity or intervals)	No	Pre- vs. post-meal responses were not compared Greater reduction in blood glucose levels after 12-weeks when meals ingested less than 2-h prior to exercise rather than more than 2-h	Not measured
Heden et al. (50)	Prospective	13 middle-aged, obese, men and women with type 2 diabetes	3-sets of 10 reps at 10RM of eight resistance/strength exercises	Yes	Not different Both pre- and post-meal activity reduced postprandial blood glucose responses	Yes Postprandial triglycerides were lower in the postprandial activity group, compared to pre-meal activity and no activity
Terada et al. (49)	Prospective	10 older aged, overweight/obese, men and women with type 2 diabetes	60 min moderate-intensity walk or interval walk	Yes	No Pre-meal activity caused lower postprandial blood glucose response than post-meal walking and no activity	Not measured

## How can sitting time be interrupted?

Recent data shows that physical inactivity accounts for more CVD-related deaths (37%) than smoking (19%), and hypertension (13%) combined, and that 15–17% of all premature deaths is attributable to low fitness (59, 60). Therefore, it is now critical that strategies to reduce inactivity are developed. Although guidelines often provide clear examples for physical activities (61), many of them are impractical. For instance, only brisk walking or using a skipping rope would be feasible activities for use as voluntary substitutes for sitting when voluntary habits like TV viewing, reading, or computer/tablet/smartphone use occur. Muscle strengthening exercises or yoga are also possible. However, sitting is often involuntary and enforced during a commute (driving cars or taking public transport) or while at school, university, or work (including office workers and delivery/public transport drivers). Accordingly, alternative approaches to reducing sitting time and inactivity during a commute and/or at the workplace are necessary.

**Figure 1:**
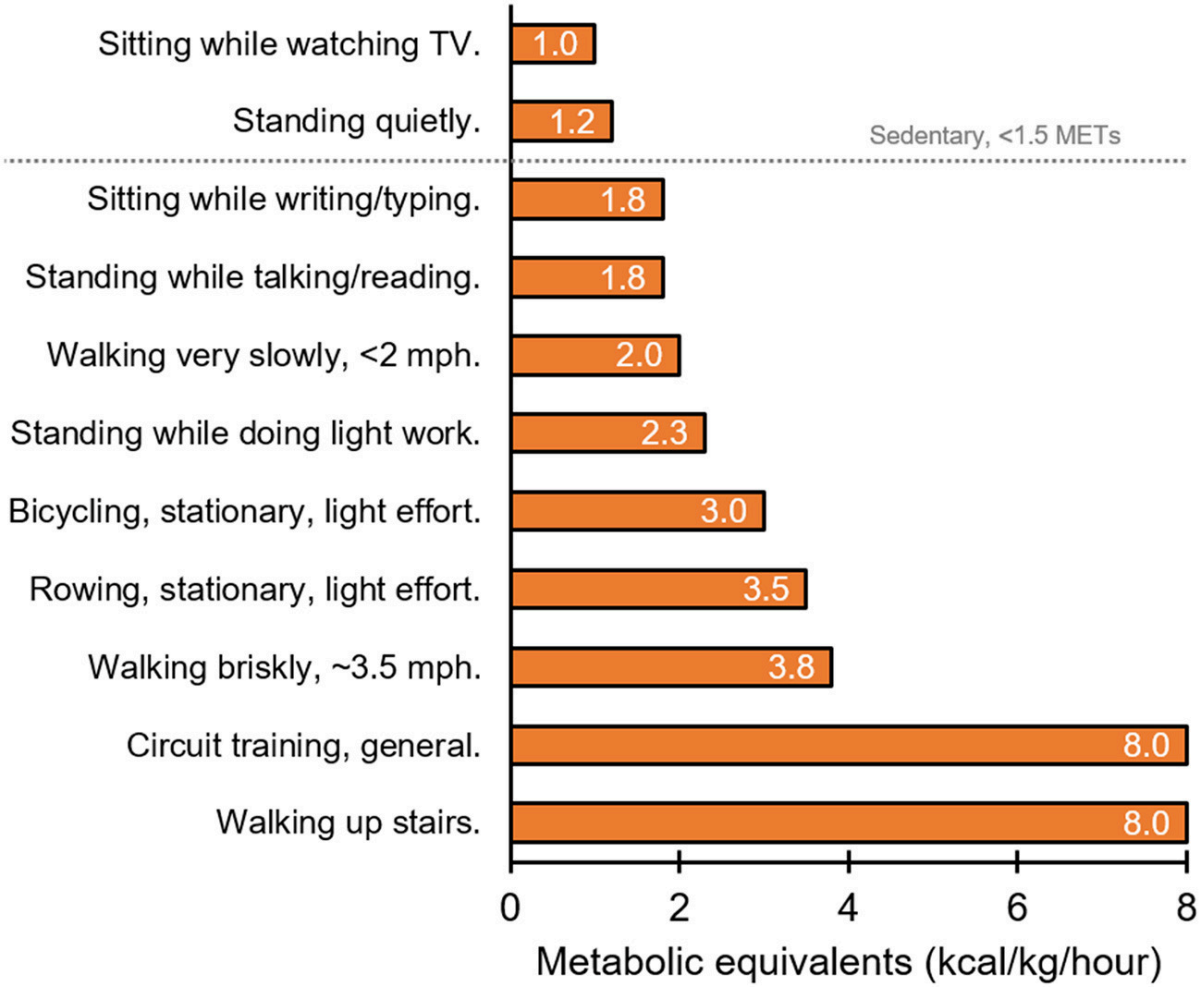
Representation of relative energy expenditure (kcal/kg/hour) during different behaviors as indicated by metabolic equivalents (METs; the ratio of the work metabolic rate to the resting metabolic rate) (70).

Previous works by Levine extensively examine the ability of different types of simple physical activities to increase daily energy expenditure above resting levels (62, 63). Not surprisingly, standing up increases energy expenditure above levels induced by sitting, while walking at incremental speeds further increases energy expenditure in a dose-dependent fashion (62). However, the magnitude of the increase in energy expenditure above basal levels that is induced by motionless activity (i.e., standing) is minor in comparison to activities that require ambulation (62, 63). For instance, Levine found that stair climbing increases energy expenditure above resting levels and expends more energy than standing in an elevator (64). Such observations have led to small-scale stair climb interventions which show some benefits to postprandial glucose control in healthy and diabetic individuals (65–69). Therefore, from an energy expenditure perspective, some form of movement to interrupt sitting would be preferable to simply standing up. This point is highlighted in Figure 1 using metabolic equivalent data extracted from the compendium of physical activities (70). That said, some evidence described above supports the use of standing alone as a means for interrupting sitting time and optimizing blood glucose control and cardiometabolic health (30–34). Further research is necessary to determine how much, when, and for whom standing is sufficient to improve blood glucose control and cardiometabolic health. Regardless, if standing is the sole activity permitted or available to interrupt sitting time, it is indeed a useful starting point.

A number of additional practical approaches to reducing sedentary time exist. Levine's work showed that greater levels of energy expenditure are induced by walk-commuting vs. drive-commuting (64). This work also found that favoring stair climbing over elevators/escalators induces greater energy expenditure (64) (Figure 1). While replacing a whole commute with a physical activity like walking is not feasible for all people, parking further from work, taking the stairs rather than an elevator, or getting off the train/bus early to integrate a brisk walk into the remainder of the commute is achievable for many. Furthermore, if taking public transport, one may consider standing up rather than sitting on the bus or train. If interrupting sitting time is not possible during a commute, physical activity may be integrated into the workday during break times or during work itself. This is important for office workers and school/university students. For instance, sitting breaks in the form of resistance exercise or body-weight circuit exercises such as squats, lunges, calf raises, press-ups, and sit-ups, can effectively improve blood glucose control and cardiometabolic risk factors (50). Such circuits, along with standing, may be favorable options since they could be undertaken at an office workstation. However, interrupting sitting time during the workday may cause distraction and reduction in work productivity/performance because ambulatory tasks like walking or cycling require information processing. Tudor-Locke (63) and MacEwen (71) have carefully evaluated the paucity of experimental studies in this area. They highlight the small samples sizes, the lack of thorough comparisons between different activities, and the heterogeneous outcomes between studies with respect to mouse use, typing speed, error rate, transcription speed, reading, and cognitive skills. It is clear, therefore, replacing workstation sedentary behavior with a physical activity must be individualized so it does not distract the worker from their work tasks. Additional strategies are also necessary and important to reduce sitting time for individuals who are unable to stand up (i.e., wheelchair users) (72).

Delivery drivers and public transportation drivers should also develop strategies to take regular breaks from sitting. Comparisons between seated workers (bus drivers) and active workers (bus conductors, postmen) in the 1950s showed increased cardiovascular morbidity in the former (73, 74), and recent data has confirmed such observations in delivery drivers (75). Furthermore, attention to people's leisure time is also essential. Epidemiological evidence shows that when people are not at work on average they sit for approximately 5 h per day watching TV, reading, or using a computer (3). Prolonged sitting time (leisure or otherwise) should be interrupted every 30-min as per diabetes prevention and treatment guidelines (11). Some data shows that home exercise can be an effective means of reducing sedentary behavior, although reduction in adherence over time is documented to necessitate additional strategies (76).

There is a clear disconnect between the knowledge of the detriments of inactivity and actual implementation of physical activity at multiple levels. Health care services have a responsibility to formulate and disseminate policy. The media have a responsibility to facilitate this dissemination to the public, with accuracy. The public also have a responsibility to empower themselves to maximize their own health. Despite these responsibilities, overwhelming evidence that scientific knowledge fails to inform public implementation emerges from objective epidemiological assessments of activity levels, where people on average are far from meeting public health activity guidelines (4, 5). However, education was an effective means to increase physical activity levels of children according to a recent meta-analysis (77). Furthermore, several groups have assessed the feasibility of workplace interventions that employers may use to keep their workforce healthy. A promising recent example is Stand Up Victoria, a multi-component intervention in Australia consisting of organizational, environmental (sit-stand workstations), and individual behavioral (i.e., face-to-face and telephone health coaching) support. In this intervention, Healy and colleagues objectively measured posture and ambulation in 231 healthy office workers (78). After 3-months intervention, overall daily sitting time and workplace sitting time were reduced, −78 min/16 h and −99 min/8 h, respectively. After 12-months, reductions in overall daily sitting time (−36 min/16 h) and workplace (−45 min/8 h) sitting time persisted, corresponding to reductions in fasting glucose and cardiometabolic risk score. The office workers primarily replaced sitting with more standing but not more ambulatory activity (78). The same group found that the reduction in workplace sitting was more effective following multi-component intervention when compared to the provision of standing desks alone (−89 vs. −33 min/8 h, respectively) over a 3-months period (79). Education alone is not always effective as the 2015 Project STAND program failed to find a significant reduction in sitting time, blood glucose or cardiometabolic risk factors in young overweight/obese adults after a 12-month education-only intervention where participants were encouraged to self-monitor and self-regulate their behavior (80). While, Aadahl and colleagues also found no reduction in sitting time after 6-months of motivational counseling, standing time was significantly increased along with improvements in cardiometabolic risk factors (waist circumference and fasting insulin) (81). From these very limited numbers of studies addressing workplace sitting time, the current meta-analysis data indicate that existing workplace interventions are not highly effective at reducing sitting time and have mixed effects at reducing cardiometabolic risk factors (82). However, interventions differ in effectiveness and the Cochrane group found that sit-stand desks were more likely to lead to less sitting than other behavioral interventions such as mindfulness training, education, or other organizational changes (83). Technology may also prove effective as randomized clinical trials have reduced sedentary time using education combined with smartphone technology to alert people when they have been inactive for a period of time (84) or by making personalized activity recommendations (85). Other studies have increased step count in the work place through cash incentives (86). However, merely wearing an activity monitor does not lead to increased physical activity in multiple studies (87, 88). Consequently, to win the war on inactivity such behavioral strategies and interventions must be optimized.

## Barriers against interrupting sitting time

To win the war on physical inactivity it is necessary to understand the health psychology of physical activity in addition to the physiology of the optimal timing between meals and physical activity. Health psychology is critical to create the optimal societal environment in which the physical activity guidelines can be achieved. As such, a huge societal shift in physical environment is required to implement physical activity into daily routines. For instance, incredible architectural foresight in major cities in Scandinavia and the Netherlands has had great impact on daily physical activity by making a commute via bicycle simple, affordable, safe, and enjoyable. In the 1970s, the Danish Government set out to develop the infrastructure needed to increase cycling as a means to reduce traffic accidents, reduce pollution, an improve health. In 2002, this culminated with a long-term “cycling policy” being established in the city of Copenhagen to increase commuting via bicycle (89). The restructuring of the physical environment to increase active transport and/or provision of sit-stand desks have also shown to be promising interventions that reduce sedentary behavior (90). The built environment interacts with health psychology to create barriers that reduce physical activity and prevent the interruption of sitting time. Regrettably, given the variability in individuals' environmental barriers against physical activity, there is no *one-size-fits-all* approach for interrupting sedentary time.

Besides the built environment, psychological barriers to energy expenditure also exist. Minimization of energy costs is biologically advantageous and is a strategy that is evolutionarily conserved (91). For example, when walking for transport, humans adopt a stride frequency, length, and width that minimizes the energy cost of the behavior (92). This minimization is learnt, linked to changes in visual perception associated with walking (93–95). Standing costs less energy than walking and sitting costs less energy than standing (Figure 1) thereby creating an incentive to avoid energy expenditure by sitting when possible. Objective data from NHANES suggest that adults sit for ≥60% of their waking hours (96). Awareness of sitting is also an issue as individuals are unware that they are sitting, and instead report the task associated with sitting rather than sitting itself (97). In support if this, when asked to categorize the behavior depicted in photographs, individuals were less likely to use posture, i.e. “sitting” vs. “standing,” to describe the task (98). Typical tasks during which individuals sit are TV viewing, computer use and/or electronic games and transportation in cars (99). Although sitting is the default behavior to minimize energy cost, tasks like TV viewing etc. dictate the behavioral choice to sit down. Therefore, it is prudent to intervene with physical activity during such tasks.

To change behavior, health education informing individuals about the risks of prolonged sitting is key (83, 90). To facilitate this, health education combined with self-monitoring of one's own behavior is a potent technique because it maintains focus on any motivation to change behavior that can result from health education (100). Self-monitoring also helps highlight to an individual a sitting behavior that they may not have been aware was occurring (97). Combing self-monitoring with goal setting has also shown promise for reducing sedentary behavior (90). Nonetheless, evidence shows that translation of motivations like self-monitoring into actual behavior change often requires volitional processes, such as reminders about motivation (101, 102) or planning about how to change a particular behavior (103–105). For example, “point-of-choice prompts” (signage at the time a healthy choice can be made) are a volitional tool that remind people about the health benefits they can accrue from increased physically active behaviors. Studies show that such prompts delivered during the day on an individual's computer can remind individuals about interruptions to sitting (106). Similarly, signs next to the office clock have encouraged individuals to break up their sedentary time when they look at the time (107).

## Going forward

It is not a new message that physical activity helps optimize blood glucose control, improve cardiovascular health, and reduce cardiovascular-related mortality, but increased awareness of the dangers of inactivity and refinement of physical activity advice is essential. Experimental evidence demonstrates that interrupting inactivity (sitting time) with physical activity breaks is a useful approach for managing blood glucose levels but recommendations concerning the optimal time to interrupt sitting do not yet exist. Since postprandial hyperglycemia is an independent cardiovascular risk factor and since we spend many hours each day in a postprandial state, timing the interruption of sitting with physical activity to minimize postprandial fluctuations in blood glucose is a sensible approach to maximize cardiometabolic health. However, randomized controlled trials determining the optimal timing between meals and activity are required and studies of this nature must examine hard endpoints like cardiovascular-related mortality. It would also be prudent for such studies to examine other cardiovascular risk factors, such as postprandial lipemia. As scientists acquire such knowledge, health psychology interventions exploring the behavioral and environmental barriers that prevent people interrupting their sedentary time must be developed. This would include consideration of the environmental barriers that influence the practicality of different activities and thus minimize the distraction from work tasks (e.g., typing, thinking, reading) or hobbies (reading, TV viewing). Furthermore, greater focus is required for increasing employers' awareness of the long-term benefits to their work force by allowing activity breaks and creating a work environment that facilitates and encourages an active workday. Such approaches will help curb the ever-increasing incidence of diabetes and thereby improve cardiovascular health, longevity, and quality of life for the increasingly inactive global population.

## Author contributions

All authors listed have made a substantial, direct and intellectual contribution to the work, and approved it for publication.

### Conflict of interest statement

The authors declare that the research was conducted in the absence of any commercial or financial relationships that could be construed as a potential conflict of interest.
